# Volatile Metabolites to Assess the Onset of Chilling Injury in Fresh-Cut Nectarines

**DOI:** 10.3390/foods13071047

**Published:** 2024-03-29

**Authors:** Michela Palumbo, Maria Cefola, Bernardo Pace, Ilde Ricci, Francesco Siano, Giuseppe Amato, Matteo Stocchero, Rosaria Cozzolino

**Affiliations:** 1 Institute of Sciences of Food Production, National Research Council of Italy (CNR), c/o CS-DAT, Via M. Protano, 71121 Foggia, Italy; michela.palumbo@ispa.cnr.it (M.P.); maria.cefola@ispa.cnr.it (M.C.); bernardo.pace@ispa.cnr.it (B.P.); ilde.ricci@ispa.cnr.it (I.R.); 2 Institute of Food Science, National Research Council of Italy (CNR), Via Roma 64, 83100 Avellino, Italy; francesco.siano@isa.cnr.it (F.S.); giuseppe.amato119@gmail.com (G.A.); 3 Laboratory of Mass Spectrometry and Metabolomics, Department of Women’s and Children’s Health, University of Padua, Corso Stati Uniti 4, 35127 Padua, Italy; matteo.stocchero@unipd.it

**Keywords:** *Prunus persica* L. Batsch, respiration rate, browning enzymes, phenolic compound, VOCs

## Abstract

Fresh-cut processing is a good strategy to enhance the commercialization of peaches and nectarines, which easily deteriorate during low-temperature storage mostly due to the occurrence of chilling injury. Although several studies have been performed to improve the shelf-life of fresh-cut stone fruit, the achievement of high-quality fresh-cut peaches and nectarines still constitutes a challenge. The present study aimed to gain insights into the evolution of the postharvest quality of fresh-cut nectarines (*Prunus persica* L. Batsch) Big Bang, cold-stored at two different storage temperatures (4 and 8 °C) for up to 10 days. Several aspects influencing the quality traits (sensory and postharvest quality parameters; the profile of phenolic and volatile organic compounds (VOCs)) were explored to predict the marketable life of the fresh-cut nectarines. The respiration rate was higher in samples stored at 4 °C, while the browning process was more evident in fruit stored at 8 °C. Partial Least Squares Regression performed on VOCs showed that samples stored at 4 °C and 8 °C presented a different time evolution during the experiment and the trajectories depended on the interaction between time and temperature. Moreover, Multiple Linear Regression analysis discovered that the 17 VOCs affected by the storage conditions seemed to suggest that no chilling injury was detected for nectarines Big Bang. In conclusion, this approach could also be used with other nectarine cultivars and/or different stone fruits.

## 1. Introduction

Regular consumption of fruits and vegetables has been universally recognized to extend protective effects against the risk of development of chronic diseases. Health benefits of food plants have been ascribed to the additive and synergistic combination of a wide range of nutrients and different bioactive components including fibers, phytochemicals, minerals and vitamins [1].

In line with this, over the last few decades, the market supply of minimally processed fruit has extensively grown in response to the demand of ready-to-use products which are particularly aligned with the modern lifestyles and eating habits of the consumers worldwide. Specifically, fresh-cut stone fruits have been demonstrated to be a good alternative to improve the commercialization of peaches and nectarines, which are economically important crops, rich in various bioactive compounds and therefore considered a useful contribution to a heathy diet. Nectarines (*Prunus persica* L. Batsch) are preferred by consumers for their pleasant flavor and sweetness. They are rich in vitamins and in several phenols and flavonoids, which contribute to their antioxidant properties [2]. However, the achievement of high-quality fresh-cut peaches and nectarines still constitutes a technical challenge and the consumption of ready-to-use stone fruit has declined over the last few years, principally because of the poor sensory traits perceived by consumers [3].

It has been demonstrated that the fruit ripening stage at harvest plays an important role in determining fresh-cut quality [4]. In particular, ethylene production, the firmness degradation rate, the sensibility to polyphenol oxidase (PPO) and peroxidase (POD) and the browning rate of the flesh [4,5,6] influence the suitability of peaches and nectarines to be minimally processed. Regardless of peaches, the possibility of processing nectarines with the skin reduces the wounding stress that occurs during postharvest of fresh-cut products [7]. Anyway, not all cultivars of nectarines are suitable for fresh-cut processing because of their different flesh softening rate: a rapid loss of firmness after harvest, which is typical of melting fruit, leads to a rapid loss of quality and consumer acceptance [8]. Moreover, the mechanical operations, including peeling, cutting, slicing and coring, cause the break of the cell walls and the release of lytic enzymes that can activate tissue degradation, triggering several physiological processes, such as ripening and senescence, which altogether lead to a substantial decrease in fruit quality [2,3]. Inappropriately, in the fresh-cut production industry, it is still commonly assumed that “if it looks good, it tastes good” [9]. Indeed, the visual appearance of the fresh-cut fruit does not always change during cold storage, and hence, the aroma profile of the processed fruit is one of the key factors of the marketability of fresh-cut nectarines. This incongruity between the apparent freshness of the product at purchase and the awareness of off-flavors at eating can decrease the consumers’ probability to repurchase the product [3]. Undesirable sensory attributes are therefore identified among the key factors of the slow progression of the fresh-cut fruit market [10], and fruit aroma, which relies on a complex interaction of several volatile organic compounds (VOCs), is considered the most important parameter in determining consumer acceptance [3].

VOCs in nectarines have been widely investigated and more than 100 components have been detected, including esters, alcohols, aldehydes, terpenes and lactones [2,3]. Peaches and nectarines’ flavor easily deteriorates during low-temperature storage [2] mostly due to the occurrence of chilling injury (CI), a physiological disorder which manifests as internal reddening, texture changes and the loss of flavor even before the development of visible symptoms [1,2,3]. Specifically, CI, which causes large economic losses during storage and transport [11], has been demonstrated to be closely related to the activities of PPO and POD [12]. Several studies have been performed to reduce CI symptoms during the storage of fresh-cut peaches and nectarines at low temperatures in order to avoid postharvest losses. Most of them have focused on the appropriateness of different varieties [13,14], the use of short-term heat treatments [15], the application of edible coatings [16,17], the enzymatic inactivation by high hydrostatic pressure processing [14,18], the effects of storage temperature and the implementation of modified atmosphere packaging (MAP) [15,19]. Storage temperature can significantly affect the respiration and the browning rate, the volatile profiles, the CI symptoms and the overall quality of fresh-cut nectarines, extending or reducing their shelf-life [4,17]. Specifically, Cozzolino et al. (2018) [19] evaluated the qualitative, the sensory and the volatile profiles of fresh and fresh-cut ‘Big Top’ nectarine slices stored at 4 °C for 8 days in active MAP or air, demonstrating that MAP preserved the overall quality throughout the storage period. Indeed, a panel of trained judges discriminated nectarine slices cold-stored in air and MAP after 8 days mostly based on visual and olfactory properties. Moreover, Partial Least Square (PLS) models applied to the VOC data and specific sensory descriptors showed that air and MAP samples could be distinguished in terms of perceived aroma owing to a mixture of odorant molecules [19].

The present study aims to assess the evolution of the quality in postharvest of fresh-cut slices of nectarines Big Bang, an early ripening cv., with an important economic value in Southern Europe [20]. This cultivar is suitable to be processed thanks to its intense redskin and yellow non-melting flesh, characterized by a subacid flavor and a low rate of ethylene production.

During the trial, fresh-cut slices of nectarines (cv. Big Bang) were cold-stored at two different storage temperatures (4 and 8 °C) for up to 10 days and several aspects influencing the organoleptic traits were explored, including sensory and postharvest quality parameters and a thorough phenolic compounds and VOCs analyses. These last analyses were performed by headspace solid-phase microextraction/gas chromatography–mass spectrometry (HS-SPME/GC-MS) to define a pool of possible volatile biomarkers that could be used to assess the quality of fresh-cut nectarines.

## 2. Materials and Methods

### 2.1. Fruit Material, Processing and Storage Condition

Nectarine fruits (*Prunus persica* L. Batsch) cv Big Bang were harvested at commercial maturity stage from a local farm located in Foggia (south of Italy) and at once transported to the postharvest laboratory of CNR-ISPA located in Foggia. At harvest, fruits showed a pH of about 3.7, a solid soluble content of about 10.1 °Brix and a titratable acidity (expressed as citric acid percentage) of about 7.6%. Nectarines were selected for absence of defects and diseases, washed in tap water, dipped in 1% sodium hypochlorite solution at room temperature for 3 min and then rinsed again with tap water to remove hypochlorite residues. All of the fruits were cut into 8 lengthwise slices around the core and packaged in 20 × 20 cm microperforated polypropylene bags (D50, Carton Pack Srl, Rutigliano, Italy; 30 μm; permeability coefficient to O_2_ and to CO_2_ of 80–95 and 250–280 cm^3^ × mm/m^2^ × bar × d at 20 °C) in a passive modified atmosphere using a packaging ma-chine (Boxer 50 GAS-Lavezzini, Fiorenzuola d’Arda, Italy). In total, 36 bags (each containing about 200 g) were prepared and stored for 10 days at 4 and 8 °C, using 6 replicates for each storage temperature. Sensory, physical and chemical parameters of fresh-cut nectarine slices were analyzed in the fresh samples and after 3, 7 and 10 days of storage. On each sampling day, all of the samples were kept for 1 h at room temperature before the analysis to make visible any cold damage due to the storage temperatures.

### 2.2. Headspace Gas Composition and Respiration Rate

The gas compositions (O_2_ and CO_2_ concentrations) inside all packages were measured on each sampling day using an infrared (CO_2_) and paramagnetic (O_2_) gas analyzer (Check-Point O_2_/CO_2_ Dansensor^®^ Mocon, Ringsted, Denmark).

The respiration rate (RR) of fresh-cut nectarines was measured at the two storage temperatures using a closed system, according to Kader (2002) [21]. In detail, for each replicate, about 200 g of the sample was put into a 3.6 L sealed plastic jar (one jar for each replicate), and the level of CO_2_ was allowed to accumulate up to 0.1% (the value of CO_2_ standard). The time taken to reach this value was detected by injecting, at regular intervals of time, 1 mL of a gas sample from the headspace of the plastic jars through a rubber septum into a gas chromatograph (p200 micro GC-Agilent, Santa Clara, CA, USA) equipped with dual columns and a thermal conductivity detector. CO_2_ was analyzed with a retention time of 16 s and a total run time of 120 s on a 10 m porous polymer (PPU) column (Agilent, Santa Clara, CA, USA) at a constant temperature of 70 °C. RR was expressed as mL CO_2_/kg × h.

### 2.3. Visual Quality and Physical Characteristics

Visual quality (VQ) and the internal browning (BR) index were measured for all of the slices in the bags by a group of 7 trained people (4 males and 3 females) on each sampling day, as reported in Pace et al. (2011) [22] and Jin et al. (2014) [23]. In particular, VQ was assessed according to a score ranging from 5 to 1, where 5 = excellent, fresh slices with no defects; 4 = very good, samples with some brown areas; 3 = fair, browned samples with low turgidity; 2 = poor, samples with severe browning and dehydrated slices; 1 = bad, samples with mold that are inedible. On this VQ scale, a score of 3 was considered the limit of marketability, while a score of 2 was the limit of edibility. Since the main symptom of cold damage in fresh-cut nectarines is the browning of the cut surface of the slices, the BR was assessed visually according to the rating scale reported by Jin et al. (2014) [23], with slight modifications. This evaluation was based on a scale from 1 to 5, where for each slice 1 = no browning; 2 = slight (up to 5% of surface affected); 3 = moderate (5–20% of surface affected); 4 = moderately severe (20–50% of surface affected); 5 = extreme (more than 50% of surface affected).

The flesh color of the fresh-cut nectarines was acquired on two opposite surface random points on the pulp of 10 slices for each replicate, using a colorimeter (CR-400-Konica Minolta, Osaka, Japan) in the reflectance mode and in the CIE L*a*b* color space. The instrument was calibrated with a white plate as standard reference (L* = 97.55, a* = 1.32 and b* = 1.87). The color was expressed as deltaE (ΔE) and deltaL (ΔL), and browning index (BI) was calculated from the primary L*, a* and b* readings, according to the equations reported by Pathare et al. (2013) [24].

The firmness of the fresh-cut nectarines was measured on 10 slices for each replicate with a puncture test using a texture analyzer (ZwickLine Z0.5-Zwick/Roell, Ulm, Germany) equipped with a stainless steel probe with a 5 mm diameter. The firmness was determined by measuring the force required by the probe to penetrate the cut surface of the slices to a dept of 10 mm, holding the surface of the nectarine pieces perpendicular to the probe [17].

Electrolyte leakage was measured according to the method reported by Jin et al. (2014) [23], with slight modifications. About 3.5 g of flesh disks (5 mm thickness × 17 mm diameter) obtained using a cork borer was placed in plastic tubes and immersed in 35 mL of distilled water. After 30 min of storage at 4 or 8 °C, the conductivity of the solution was measured using a conductivity meter (Cond. 51+-XS Instruments, Carpi, Italy). Then, the tubes containing the samples and solution were frozen at −20 °C, and after 48 h, the final conductivity was measured after thawing and considered as total conductivity. Electrolyte leakage was calculated as the percentage ratio of initial conductivity over total conductivity.

### 2.4. Antioxidant Activity, O-Quinones, Total Carotenoids and Total Sugars

The antioxidant activity was assessed as previously reported by Fadda et al. (2016) [25] at harvest (0 days) and at the end of storage (10 days). In detail, 50 μL of the extract obtained as reported in [26] was combined with 950 μL of a solution of 0.1 mM DPPH in ethanol, and the mixture was incubated for 30 min at room temperature in the dark. The absorbance of the test solutions was read at 515 nm and compared to that of the non-inhibited DPPH. Data were referenced to a calibration curve which was built with Trolox (82–625 μM, R^2^ = 0.99) and the antioxidant activity was expressed as milligrams of Trolox per 100 g of fresh weight (fw).

The same supernatant was directly used to measure the o-quinones at a wavelength of 437 nm [26]. The result was expressed as the absorbance for 5 g of fw.

For the determination of total carotenoids, 5 g of chopped nectarine slices was homogenized in 20 mL of acetone/water (80:20 *v*/*v*) and then centrifuged at 6440× *g* for 5 min. To remove all pigments, the extraction procedure was repeated 3 times, and the extracts were combined. The absorbance (Abs) was measured at three wavelengths, 663.2, 646.8 and 470 nm, immediately after the extraction. Carotenoids were calculated using the following formula:

[(1000 × Abs 470) − (1.82 × Ca) − (85.02 × Cb)]/198(1)

where Ca = (12.25 × Abs 663.2) − (2.79 × Abs 646.8), and Cb = (21.50 × Abs 646.8) − (5.10 × Abs 663.2) [27]. The results were expressed as mg of carotenoids per kg of fruit on fw.

The content of total sugars was determined using a phenol-sulfuric colorimetric method [28]. Briefly, 5 g of chopped nectarine slices was homogenized with 20 mL of 95% ethanol for 2 min and centrifuged at 6440× *g* for 5 min. The extracts, properly diluted, were used for the determination of the color development at 490 nm. Glucose was used as standard (y = 78.777 (±3.60) x + 4.1582 (±1.32); R^2^ = 0.99) and sugar content was expressed on a fw basis and reported as mg glucose/g.

### 2.5. Polyphenol Oxidases (PPO) and Peroxidases (POD)

PPO and POD activity was determined according to the method reported by Cefola et al. (2012) with slight modifications [29]. Nectarine slices were cut into small pieces using a stainless steel knife. A 10 g subsample was homogenized for 1 min in ice with 20 mL of chilled phosphate buffer (0.05 M, pH 6.2) and 30 g/L of polyvinylpyrrolidone PVPP. The mixture was filtered through two layers of Miracloth and centrifuged at 15,000× *g* for 15 min at 4 °C. The supernatant was recovered and kept in ice until being assayed (within 5 h).

For the PPO assay, the enzyme activity was determined by using 15 mM chlorogenic acid in 0.05 M potassium phosphate buffer, pH 6.2, following the oxidation of 15 mM chlorogenic acid at 410 nm for 2 min. One unit of PPO activity was defined as a 0.001 absorbance change per minute per gram of fw under the above described assay conditions.

POD activity in the supernatant fractions was determined with 15 mM chlorogenic acid as a reducing substrate in a reaction mixture containing 0.1 M potassium phosphate buffer, pH 5.0, and 30 mM H_2_O_2_. The oxidation of chlorogenic acid was assessed by observing the absorbance increase at 470 nm for 2 min. One unit of PPO activity was defined as a 0.001 absorbance change per minute per gram of fw under the above assay conditions.

### 2.6. Phenylalanine Ammonia-Lyase (PAL)

PAL activity was determined using a modified version of the method reported by Gao et al. (2018) [30]. In detail, nectarine slices were cut into small pieces and a 5 g subsample was homogenized for 1 min in ice with 15 mL of chilled 0.2 M boric acid–NaOH buffer (pH 8.8) containing 5 mM 2-mercaptoethanol and 30 g/L of polyvinylpyrrolidone (PVP). The mixture was filtered through two layers of Miracloth and centrifuged at 15,000× *g* for 10 min at 4 °C. The supernatant was recovered and kept in ice until being assayed. The reaction mixture for the PAL assay consisted of 0.5 mL enzyme extract, 3 mL 0.05 M boric acid–NaOH buffer (pH 8.8) and 0.5 mL 20 mM L-phenylalanine. The absorbance at 290 nm was read and then the reaction mixture was incubated for 60 min at 40 °C. After the incubation period, the reaction was stopped by adding 0.1 mL 6 M HCl, cooled at room temperature and measured at 290 nm. The change in absorbance (ΔA290) was calculated for all samples. One unit of PAL activity was defined as the amount of enzyme that causes an increase in absorbance by 0.01 at 290 nm in 60 min. A standard curve for trans-cinnamic acid at various concentrations in ethanol was obtained adding 100 µL of 6 M HCl and reading the absorbance at 290 nm.

### 2.7. Volatile Organic Compounds Analysis

#### 2.7.1. Sample Preparation and HS-SPME Extraction

VOCs were extracted according to the HS-SPME procedure described in [19] using the DVB/CAR/PDMS (50/30 µm) fiber, with an extraction temperature and an extraction time of 40 °C and 20 min, respectively. Nectarine samples were prepared by cutting several slices of fruit from the same package and collecting 1 g from the whole sample to obtain a representative sample from each bag. Briefly, 1 g of chopped nectarine slices was put in a 20 mL HS vial with a screw cap containing 0.5 g of NaCl and 5 mL of ultra-pure water from a Milli-Q system. Additionally, 10 µL of 3-octanol (0.4 µg/mL) was added as the internal standard (IS). The vials, closed with a PTFE–silicone septum, were placed at 40 °C in the instrument dry block-heater for 20 min. Afterwards, the fiber was mechanically inserted into the vial’s septum for 20 min for the VOCs adsorption onto the SPME fiber surface. The extraction and injection steps were automatically performed by an autosampler MPS 2 (Gerstel, Mülheim, Germany).

#### 2.7.2. GC-MS Analysis

VOCs were analyzed by an Agilent 7890A gas chromatography device (7890A, Agilent Technologies, Santa Clara, CA, USA) interfaced with a 5975A mass spectrometer (5975A, Agilent Technology, Santa Clara, CA, USA). The fiber was inserted into the injection port of the GC for 10 min and volatiles were desorbed at 250 °C and directly transported to a capillary column HP-Innowax (30 m × 0.25 mm × 0.5 μm). For separation, the oven temperature was initially held at 40 °C for 5 min, then ramped up to 150 °C at 4 °C/min and maintained for 5 min. Helium was used as the carrier gas at a flow rate of 1.5 mL/min. The ion source and quadrupole temperatures were set at 230 °C and 150 °C, respectively. Mass spectra were acquired in electronic impact (EI) mode at 70 eV and mass spectra were scanned in the range *m*/*z* 30–300 at 2.7 scans/s. Volatile components were detected in pulsed splitless mode. Metabolites were identified or tentatively identified by comparing their mass spectra with the available libraries (NIST, version 2005; Wiley, version 2007) and by matching their retention times with an in-house developed retention time library based on commercial standards. Moreover, identification of VOCs was also accomplished by comparing their retention indices (RIs) (as Kovats indices), calculated in relation to the retention time of a C_8_–C_22_ n-alkanes series with linear interpolation, with those of authentic compounds or the literature data. Semi-quantitative data of each metabolite (Relative Peak Area, RPA%) were measured with respect to the peak area of 3-octanol (IS). Areas of the identified metabolites were calculated from the total ion current (TIC). Samples were analyzed in triplicate and blanks were also acquired.

### 2.8. Extraction of Phenolic Compounds and HPLC-DAD Analysis

Nectarine samples (1.0 g) were homogenized with 10 mL of 80% aqueous methanol (*v*/*v*) in an ultrasonic bath for 30 min. Samples were then subjected to centrifugation (2500× *g* at 4 °C for 10 min) and filtration using 0.22 μm PVDF disposable syringe filters (Millex, Millipore, Badford, MA, USA). A total of 1 mL the supernatant was collected and concentrated in a Savant speedvac then reconstituted to 1 mL with 0.1% trifluoroacetic acid (TFA) and stored at −20 °C until further analysis.

Phenolic compounds were separated using a modular chromatographer HP 1100 (Agilent Technologies, Paolo Alto, CA, USA) equipped with a 250 × 2.1 mm i.d. Jupiter C18 reverse-phase column with a 4 mm particle diameter (Phenomenex, Torrance, CA, USA), and were maintained at 37 °C using a thermostatic oven. Separations were carried out at a 0.2 mL min^−1^ constant flow rate, applying the following gradient of the solvent B (ACN/0.1% TFA): 0–4 min, 0% B; 4–14 min, 0–14% B; 14–30 min, 14–28% B; 30–34 min, 28% B; 34–42 min, 28–60% B; 42–45 min, 60–80% B; 45–50 min, 80–100% B. Solvent A was 0.1% TFA in HPLC grade water. For each analysis, 100 μL of the extract was injected. A diode array detector (DAD) was used to record the UV–vis spectra every 2 s in the 190–650 nm range. The HPLC separations were monitored by recording the λ = 520, 360, 320 and 280 nm wavelengths.

Data were processed using the ChemStation software version A.10 (Agilent Technologies, Santa Clara CA, USA). Compounds were identified by the convergent information of retention time order. Peak assignment of chlorogenic acids, cyanidin-3-O-glucoside and rutin (all purchased from Merck-Sigma, Milan, Italy) was validated by chromatographic comparison with authentic standards. Phenolic compounds were quantified by generating calibration curves from standard solutions (R^2^ > 0.99) prepared at six different concentrations (5–250 mg/kg) in methanol and subsequently diluted (10 times) with 0.1% trifluoroacetic acid before injection. Each sample was analyzed in triplicate and peak area values were averaged. The results were expressed as mg/kg FW.

### 2.9. Statistical Data Analysis

The experiment was a longitudinal study where biological samples were investigated at 3 time points (factor “time” with values 3, 7 and 10 days) considering 2 different storage conditions (factor “storage” with levels equal to 4 °C and 8 °C). For each design cell, 3 biological samples were investigated.

As a preliminary data analysis, data of the fresh fruit were compared with those of the stored slices using the Dunnett’s test, controlling the false discovery rate by using the Benjamini–Hochberg method [31]. Thus, the experimental design was explicitly taken into account in data analysis. Specifically, Partial Least Squares Regression (PLS) was adapted to study the designed experiments following the approach introduced in Stocchero (2023) [32].

The number of score components used was assessed by testing the significance of the eigenvalues, calculated by solving the PLS problem. Selectivity Ratio (SR) was used as a score to select the most important features of the model. A significance level of 0.05 was assumed in statistical testing. For a comprehensive data analysis, Multiple Linear Regression (MLR), controlling the false discovery rate at a level of 0.05 by using the Benjamini–Hochberg method [31], was applied by regressing the data on the design matrix. The relationships between responses during the storage were investigated by Pearson’s correlation-based analysis.

Data analysis was performed by in-house R-functions implemented using the R 4.0.4 platform (R Foundation for Statistical Computing, Vienna, Austria).

## 3. Results and Discussion

### 3.1. Quality Changes in Fresh-Cut Nectarines during Storage

**Figure 1:**
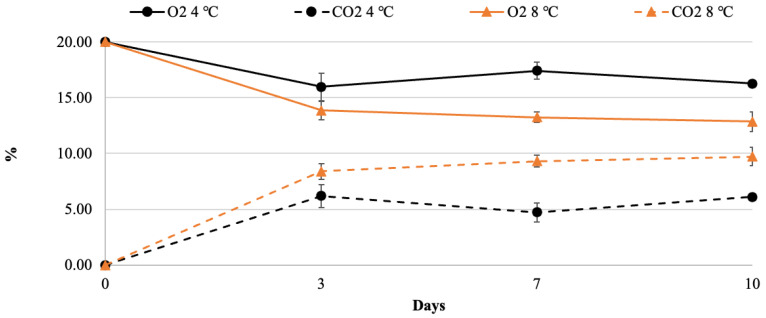
Changes in O_2_ and CO_2_ concentrations in fresh-cut nectarine slices packaged in polypropylene bags during storage at 4 or 8 °C.

The concentration of O_2_ and CO_2_ in the PP bags containing the fresh-cut nectarines changed during the postharvest storage, as reported in Figure 1. Nectarines slices packaged in a passive atmosphere showed a progressive decrease in O_2_ and a parallel increase in CO_2_.

The percentage of O_2_ and CO_2_ displayed a similar trend at both storage temperatures (4 and 8 °C) and each gas reached the equilibrium after 3 days of storage (Figure 1). In particular, O_2_ showed a lower decrease in the samples at 4 °C compared to those at 8 °C (achieving values of about 15.9 and 13.8%, respectively, at the equilibrium), while CO_2_ concentration increased up to 6.2 ± 1.4 and 8.4 ± 0.7% in nectarines stored at 4 and 8 °C, respectively. The slight differences in the gas composition inside the bags could be explained by the different respiration of the fruit stored at two different temperatures. It was reported that in peaches and nectarines, a CO_2_ concentration above 5% during storage can delay the beginning of the chilling injury [4,33]. According to the literature, our data can indicate that the CO_2_ levels reached at the equilibrium for both storage temperatures were suitable for keeping the postharvest quality of nectarines.

### 3.2. Volatile Compounds of Fresh-Cut Nectarines during Storage

Overall, the HS-SPME/GC-MS analysis of the nectarine slices cold-stored at two different temperatures (4 and 8 °C) allowed us to detect and semi-quantify 46 volatile compounds which could be clustered in different chemical classes, including esters (15), ketones (4), aldehydes (9), alcohols (10), terpenes (3), acids (2) and others (3), as summarized in Table S1.

Most of the metabolites listed in Table S1, which also reports the VOCs’ abbreviation code, the experimental and literature Linear Retention Index (LRI) and the identification method, have already been reported in the literature in different cultivars of nectarines [2,3,34,35,36,37,38].

As a preliminary data analysis, a Dunnett’s test was performed to evaluate the effect of the storage conditions (4 and 8 °C up to 10 d) on the volatiles detected in the nectarine slices, with respect to the fresh samples. Since 46 VOCs were observed, the false discovery rate was controlled by using the Benjamini–Hochberg method, assuming a level of δ 0.01. Except for pentanoic acid (Ac1), significant differences in all volatiles were detected across the different temperatures and storage days (Table 1).

In detail, alcohols, mainly 1-hexanol (Al3), *trans*-3-hexen-1-ol (Al5) and *trans*-2-hexen-1-ol (Al6), were the most representative group in the fresh fruit, accounting for 62.3% of the total volatile content (Table 1). Esters, primarily hexyl acetate (E7), *trans*-3-hexenyl acetate (E8) and *cis*-2-hexenyl acetate (E9), were the second most abundant chemical class, representing 19.0% of the total VOC profile, followed by aldehydes, mostly 2-hexenal (Ald 4), which accounted for 17.4% of the total volatiles (Table 1). Finally, ketones and terpenes were found in low amounts (about 1.0 and 0.4% of the total VOC content, respectively) (Table 1). Significant changes were detected in the volatile profile of all sliced nectarine samples following storage at the two different temperatures, as new compounds were produced and considerable losses occurred (Table 1). Moreover, consistent with Zhang et al. (2022), the two storage temperatures had different impacts on nectarine VOCs during the postharvest storage (Table 1).

**Figure 2:**
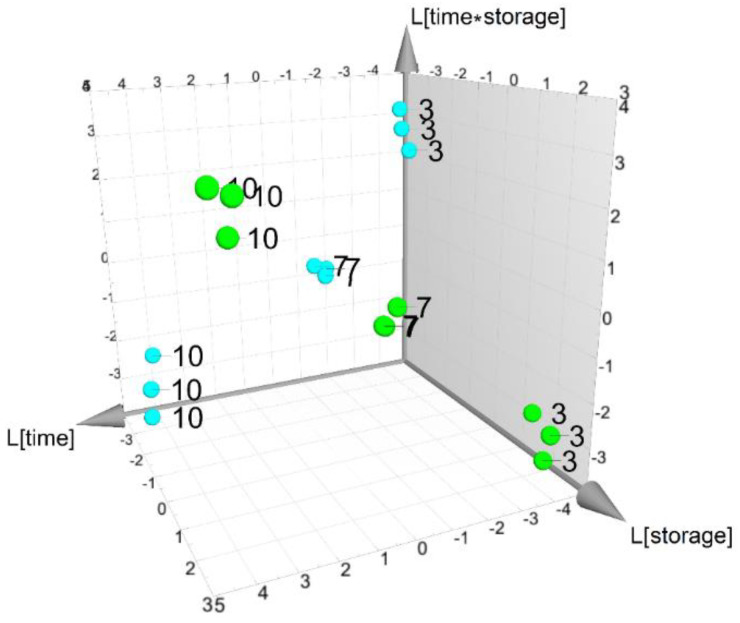
VOC dataset: score scatter plot of PLS-based model. Samples stored at 4 °C are reported as green spheres, while samples at 8 °C as cyan spheres. Labels are used to indicate time points.

To perform a data analysis of the VOCs during the time of storage, considering the three time points (3, 7 and 10 days) and the two storage conditions (4 and 8 °C), a dataset composed of 46 VOCs and 18 observations was obtained. Based on a design matrix with an interaction term between “time” and “storage”, a PLS model with three latent variables, R^2^_time_ = 0.983 (*p* < 0.01), V^2^_storage_ = 0.790 (*p* < 0.01) and R^2^ _storage time_= 0.988 (*p* < 0.01), was obtained. All factors resulted in being significant. The score scatter plot of Figure 2 shows that samples stored at 4 °C and 8 °C presented a different time evolution during the experiment and the trajectories depended on the interaction term. Investigating the spectrum of the SR score, five VOCs (E1, E5, E8, E10 and Al9) resulted in being affected by the factor “storage”.

**Figure 3:**
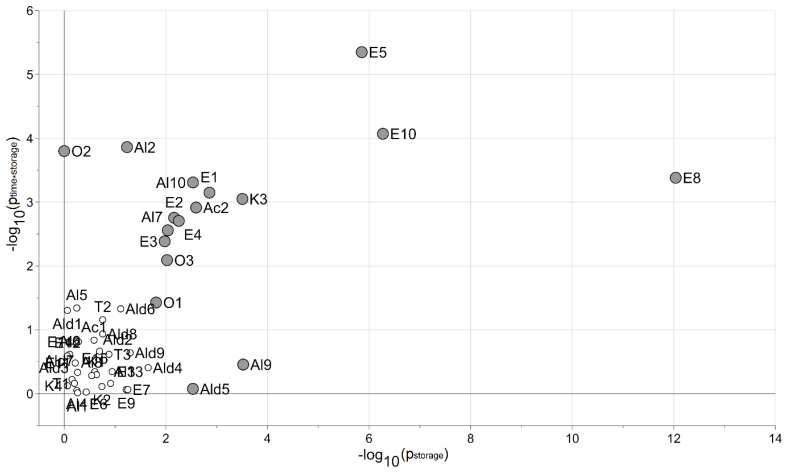
The VOC dataset: the MLR-based analysis. The VOCs associated with the effect of the storage condition are reported as gray circles, while VOCs not associated with the storage conditions are reported as white circles. The negative logarithm of the *p*-values of the factor “storage” (horizontal axis) and of the interaction term (vertical axis) are used.

**Figure 4:**
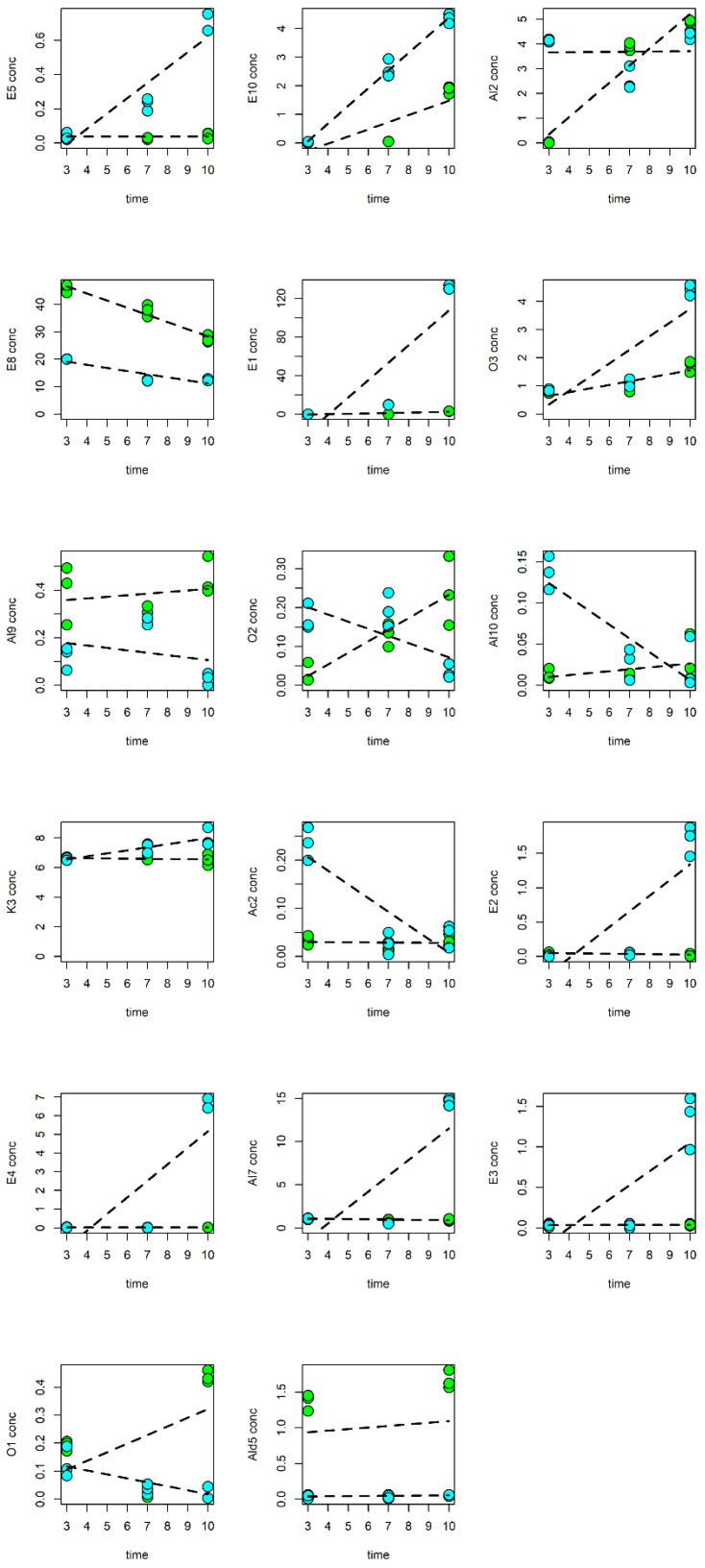
The trend profiles of the VOCs significantly affected by the storage conditions. The samples stored at 4 °C and at 8 °C are reported as green and cyan circles, respectively.

Moreover, the MLR-based analysis, performed considering a model with the interaction, discovered 17 VOCs affected by the storage conditions. The set of VOCs were included in the compounds selected by the PLS analysis. The results are reported in Figure 3, while the trend profiles of the 17 selected VOCs are represented in Figure 4.

Specifically, total alcohols showed a decreasing trend at the two temperatures compared to the fresh fruit (Table 1). The amount of hexanol (Al3), the most abundant alcohol in the fresh nectarines (25.2% of the total VOC content), gradually decreased at 8 °C (12.4, 7.6 and 5.3% at 3, 7 and 10 d, respectively), while it presented a fluctuating trend at 4 °C, as it sharply declined at 3 d (12.0%) then increased at 7 d (14.2%) and decreased again at 10 d (7%). In any case, the amount of Al3 was always lower with respect to the fresh samples, regardless of the storage temperature (Table 1; Figure 4). A similar behavior was shown by *trans*-2-hexen-1-ol (Al6), the second most abundant alcohol in the fresh fruit (23.9% of the total VOC content), as it presented a decreased concentration in nectarine slices stored both at 4 °C and at 8 °C with respect to the untreated samples (Table 1; Figure 4). These findings are partially consistent with those reported by Zhang et al. (2022) [2], who observed a decrease in hexanol throughout storage at 5 °C and an increase in *trans*-2-hexen-1-ol up to 14 d in nectarines stored at 8 °C [2].

Compared to the fresh fruit, total aldehydes showed an increasing trend at both temperatures (Table 1). In particular, *trans*-2-hexenal (Ald 4), the main aldehyde in fresh nectarines (11.2% of the total VOC content), and hexanal (Ald1), the second most abundant compound of this class in the untreated fruit (5.4% of the total VOC content), presented a trend similar to that exhibited by the total aldehydes, as they showed higher amounts on each sampling day at both temperatures with respect to the fresh samples (Table 1, Figure 4).

Chilling injury-inducing temperature (5 °C) has been reported to suppress the production of total aldehydes in nectarines during storage [2], while an increase in C6-aldehydes is commonly associated with a typical response to the mechanical damages and to the tissue disruption driven by the lipoxygenase (LOX) activity [38,39]. Furthermore, C6-aldehydes are involved in the signaling network subsequent to the activation of plant defense triggered by the mechanical injury in plant tissues [40]. The increasing trend of the amount of total aldehydes during storage, regardless of the used temperature (Table 1), seems to suggest that these volatiles are more influenced by postharvest processing rather than by the storage temperature, indicating that this specific cv could be not affected by CI. In this regard, several previous reports have observed that the influence of cold storage on the aldehyde content in nectarine samples is strongly cultivar-dependent [2,37,41].

Total esters showed a fluctuating trend during storage regardless of the temperatures. At 4 °C, a lower content of total esters was observed at 3 and 10 d (10.2 and 8.5%, respectively) compared to the fresh fruit (19.0%), while a significantly higher amount was detected at 7 d (35.8%). On the contrary, total esters in fresh-cut nectarines stored at 8 °C were always lower than in the fresh samples, even if after a substantial decrease after 3 d (5.5%), the amount increased at 7 and 10 d (10.0 and 14.3%, respectively) (Table 1). The content of the principal ester, *trans*-3-hexenyl acetate (E8, 8.9% of the total VOCs), was always lower compared to the fresh fruit at both temperatures during the cold storage, while the amount of hexyl acetate (E7) and *cis*-2-hexenyl acetate (E9) was lower with respect to the fresh samples on each sampling day only at 8 °C. On the contrary, at 4 °C, the concentration of E7 and E9 followed a trend similar to that shown by the total ester amount, presenting higher values at 7 d (16.2% for E7 and 15.9% for E9) with respect to the control samples (Table 1; Figure 4). In any case, E7, E8 and E9 exhibited significantly lower contents in nectarines stored at 8 °C, with respect to those at 4 °C, during the entire storage period (Table 1). Moreover, the amounts of ethyl caprylate (E10) and γ-caprolactone (O3) increased at both temperatures from the 3rd to the 10th day, and at the end of the storage period, they were always higher at 8 °C compared to at 4 °C (Figure 4). Similarly, the contents of five ester compounds, including ethyl acetate (E1), ethyl propionate (E2), propyl acetate (E3), 2-methylpropyl acetate (E4) and ethyl butyrate (E5), and of the alcohol 1-octen-3-ol (Al7) remained constant at 4 °C, while they increased at 8 °C, showing higher values at this temperature compared to at 4 °C at the end of the storage period. On the other hand, the content of *cis*-3-hexen-1-ol acetate (E8) decreased during preservation at both temperatures, but it was higher at 4 °C compared to at 8 °C at the end of storage (Figure 4). These results are partially consistent with those reported by Zhang et al. (2022), who detected a lower production of esters during the postharvest storage of nectarines, but a higher content of E8 in fruit stored at 8 °C compared to at 5 °C during the entire storage period.

The production of ethyl and acetate esters has been ascribed to fruit ripening and senescence [3], since nectarines are climacteric fruit. This could explain the identification of ethyl acetate (E1) at 10 d at 4 °C and from 7 d at 8 °C (Table 1) and the observation of ethyl propionate (E2), propyl acetate (E3), 2-methylpropyl acetate (E4) and ethyl butyrate (E5) only at the end of the storage period at 8 °C (Table 1).

Esters and lactones, considered the main contributors to the typical peach-like aroma of fruit, are known to increase throughout fruit ripening and during cold storage at non-chilling injury-inducing temperatures [37]. Inversely, in agreement with previous reports, chilling-injured fruits have been reported to present a decreased production of esters and lactones, but an accumulation of aldehydes and alcohols throughout storage [2,37]. Specifically, CI occurrence, which is linked with a lower production of ethylene, has been described to reduce the activity of two enzymes: acyl-CoA oxidase (ACX), the first rate-limiting enzyme for the synthesis of lactones through the β-oxidation pathway, and alcohol acyl-transferase (AAT), which is involved in the reaction of esterification between alcohols and acyl-CoAs for the production of esters through the lipoxygenase (LOX) pathway [2,37]. Consequently, in chilling-injured nectarines, the decrease in esters can stimulate the accumulation of aldehydes and alcohols; esters, in fact, are produced from alcohol and aldehyde precursors through the LOX pathway [2,37]. As our findings are in contrast with this trend, they could suggest once again that nectarines Big Bang cv are not affected by chilling injury.

Anyway, it is important to underline that some alcohols, namely, *cis*-2-penten-1-ol (Al2), 1-octanol (Al9) and 2-furanmethanol (Al10), and the aldehyde Ald5 (octanal) increased their concentration at 4 °C during the period of storage, presenting a higher amount on the 10th day at 4 °C compared to at 8 °C (Figure 4). The accumulation of these compounds can suggest the possible onset of CI in the nectarine samples at 4 °C compared to at8 °C [36].

Total terpenes, essential for the perception of the typical fruity, alcoholic and fresh floral notes in stone fruits, showed a decreasing trend with respect to the fresh samples (5.2% of the total VOC content), regardless of the storage temperature, as previously reported (Table 1; Figure 4) [2]. Limonene (T1) was detected only in the fresh fruit, while linalool (T2), previously acknowledged as an abundant volatile in the skin and flesh of peaches [2], sharply declined at 3 d at both 4 and 8 °C (0.5 and 0.3, respectively) and was absent afterwards (Table 1). In parallel to esters and lactones and consistent with our results, linalool concentration has been described to increase throughout peach ripening and to significantly decrease during the cold storage of stone fruit [2,37,41,42]. Finally, α-farnesene (T3) was observed up to 7 d at both temperatures. Consequently, at the end of the storage period, no terpene compound was detected (Table 1).

### 3.3. Phenolic Compounds of Fresh-Cut Nectarines during Storage

Several simultaneous events may regulate the quantitative changes in phenolic compounds during fruit storage, including (i) dehydration, which is expected to indiscriminately enhance to variable extents the content of individual compounds; (ii) oxidation, which can selectively decrease the concentration of some metabolites in relation to their susceptibility to spontaneous and enzyme-assisted oxidative degradation; (iii) active metabolism, which can reduce the amount of precursors and increase the final products of specific pathways. The concentrations of individual polyphenols estimated by HPLC-DAD analysis at several post-storage time points for fresh and stored fresh-cut nectarine samples are reported in Table 2. A Dunnett’s test controlling the false discovery rate at a level of 0.05 was performed to assess the effect of storage at two different temperatures (4 and 8 °C) for up to 10 d on the phenolic compounds identified in the nectarine slices, with respect to the fresh samples. This test showed that significant differences in all components were detected throughout the storage period at different temperatures (Table 2).

No clear pattern appeared across the variations in phenolic compound content detected throughout storage, other than for rutin (P5), which was the only compound significantly affected by the storage temperature, as highlighted by the MLR-based analysis performed on the dataset based on the concentration of five polyphenolic compounds reported in Table 2. Moreover, no effect of the factor “time” was observed.

**Figure 5:**
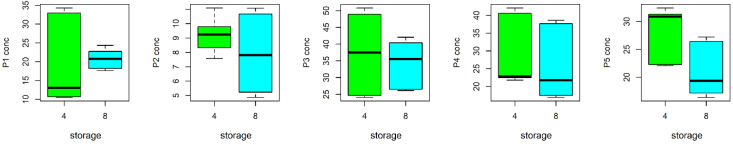
Box plots representing the distributions of the polyphenolic compounds with respect to the storage condition. P1 (neochlorogenic acid); P2 (cryptochlorogenic acid); P3 (chlorogenic acid); P4 (cyanidin-3-O-glucoside); P5 (rutin).

The distributions of the concentration of the five polyphenolic compounds are represented in the box plots of Figure 5.

The ripening process of fruit can be prolonged even during storage at refrigerated temperatures. In the subsequent stages of maturation, after a significant increase in polyphenols, these compounds may decrease due to the oxidation process activated by the polyoxidases [43]. Not all phenol components undergo the oxidation action to the same extent, and this could justify the significant variability seen for rutin (P5) underlined by the MLR-based analysis (Figure 5).

### 3.4. Analysis of Other Compounds in Fresh-Cut Nectarines during Storage

No significant effects of storage conditions were detected for total sugars, total carotenoids and soluble *o*-quinones.

### 3.5. Data Analysis of the Responses of Fresh-Cut Nectarines during Storage

**Figure 6:**
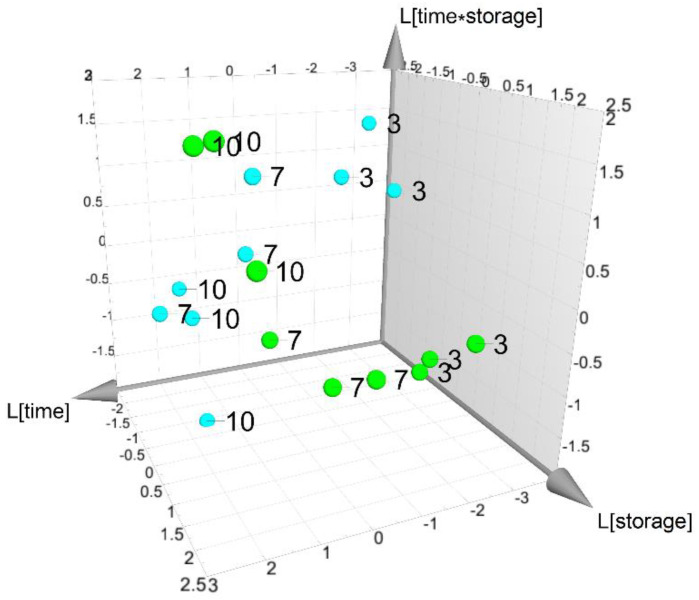
The dataset of the responses: a score scatter plot of the PLS-based model. The samples stored at 4 °C are reported as green spheres, while samples at 8 °C are shown as cyan spheres. Labels are used to indicate the time points.

**Figure 7:**
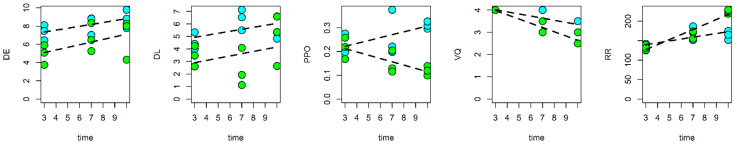
The profiles of the responses (DE, DL, PPO, VQ and RR) significantly affected by the storage condition. The samples stored at 4 °C and at 8 °C are reported as green and cyan circles, respectively.

Considering a design matrix with interaction, the PLS-based analysis of the dataset composed of 11 responses generated a model with three latent variables, R^2^_time_ = 0.937 (*p* < 0.01), V^2^_storage_ = 0.790 (*p* < 0.01) and R^2^_time*storage_ = 0.810 (*p* < 0.01). Time, storage condition and the interaction term were significant. Investigating the score scatter plot of Figure 6, it is possible to note that the trajectories of the samples depend on the storage condition. The analysis of the SR spectrum highlighted that the responses of the parameters VQ, ΔL, RR and PPO were affected by the storage condition. The MLR-based analysis discovered that the responses of PPO, VQ, ΔE, RR and ΔL were significantly affected by the factor “storage”. The profiles of the selected responses are represented in Figure 7.

In detail, ΔE and ΔL showed a similar trend: they increased during the storage period, with values higher in samples stored at 8 °C (8.8 ± 1.0 and 5.6 ± 0.9, respectively) compared to at 4 °C (6.8 ± 2.0 for DE and 4.8 ± 2.0 for DL) (Figure 7). Wen et al. (2018) [44] suggested that the L* value and its variation (DL) during storage may be an effective indicator of color darkening in fresh-cut produce. Moreover, the DE value represents the difference in the color of samples during storage compared to the color of the samples at harvest. The results of the present study suggest that storage at 4 °C preserved the color of fresh-cut nectarine slices better compared to storage at 8 °C.

In regard to VQ, a general decrease in this parameter on fresh-cut nectarines was recorded at both temperatures during storage, reaching the marketable limit (score 3) after 7 and 10 days in samples stored at 4 and 8 °C, respectively (Figure 7). The loss in VQ is usually related to browning, color changes and wilting of the cut surface of the slices [17]. In this study, even if no evident CI symptoms appeared on the cut surface of nectarine slices, slight differences in the sensory VQ were perceived by the panelists among the two storage temperatures, as demonstrated by the color data.

The PPO activity along the storage showed an opposite trend for the two temperatures (Figure 7). In particular, in samples stored at 4 °C, the PPO activity slowed down until the 10th day; on the contrary, it increased in fresh-cut nectarines during storage at 8 °C. This suggests that in the fruits stored at 8 °C, the rupture of cell structure has probably happened, with a consequent increase in the solubility of PPO and its easier contact with phenolic substrates [45]. These results agree with the color data (∆E and ∆L) obtained for samples stored at 8 °C, which showed greater changes in the color of the pulp at the end of storage.

Finally, the RR just after slicing was 12.18 ± 0.87 mL CO_2_/kg h and increased more during storage in samples at 4 °C than in those stored at 8 °C, reaching values of 224.34 ± 4.99 and 164.11 ± 11.93 mL CO_2_/kg h, respectively. The respiration of fruits is usually considered the most relevant index to express the physiological activity, as well as the storability of a product [46]. A reduced RR during storage may be attributed to a lower metabolic activity of the samples stored at 8 °C compared to those at 4 °C. Most likely, fresh-cut nectarines at 4 °C started an intense physiological activity due to the low temperature stress, even if no evident symptoms typical of CI have developed on the cut surfaces of nectarine slices. In fact, peaches and nectarines stored within 2.2–7.6 °C are more susceptible to this disorder [33].

**Figure 8:**
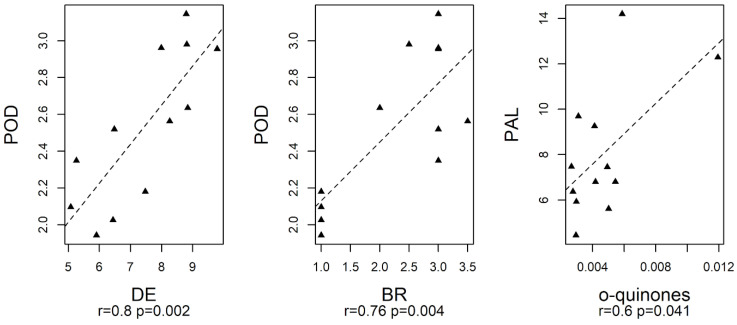
Correlation analysis based on Pearson’s correlation. Black triangles represent experimental measures whereas dashed lines show linear regression lines; r and *p* are Pearson’s correlation coefficient and its *p*-value, respectively.

The relationships among the enzymes involved in browning (PPO, POD and PLA) and DE, DL, BR, and *o*-quinones were investigated by Pearson’s correlation assuming a significance level of 0.05 (Figure 8). In detail, POD showed a significant positive correlation with DE and BR, while PAL showed a positive correlation with *o*-quinones, confirming the role of the enzymes in browning development. PAL activity promotes an increment in the concentration of phenolic compounds, which are substrates for oxidative enzymes, such as PPO and POD, that in the presence of O_2_ and H_2_O_2_ produce *o*-quinones, which polymerize to brown pigments and are responsible for the browning of the cut surfaces of fresh-cut produces [44,47,48].

## 4. Conclusions

The present study proposed a valid approach to evaluate the occurrence of the chilling injury in fresh-cut nectarines by observing the changes in the VOC profiles throughout storage at two temperatures (4 and 8 °C). The correlation analysis, carried out on the quality data, including color parameters and *o*-quinones, allowed us to confirm the role of the enzymes PAL and POD in the browning process of fresh-cut nectarines, which was more evident in fruit stored at 8 °C compared to at 4 °C. Moreover, the climacteric behavior of nectarines was responsible for the browning process and also affected the modifications observed in the volatile patterns, with the accumulation of esters and aldehydes in fruit stored at 8 °C.

On the other hand, a higher amount of specific volatile markers, including *cis*-2-penten-1-ol, 1-octanol, 2-furanmethanol and octanal, was observed at the end of the storage period at 4 °C compared to at 8 °C, suggesting a possible onset of CI in the nectarine samples only at 4 °C. The respiration rate confirmed this observation, since it was higher in samples stored at 4 °C, creating a stressful condition for the fruits.

In conclusion, the suggested approach, which has been revealed to be useful to evaluate the beginning of the occurrence of chilling injury in fresh-cut nectarines Big Bang stored at 4 °C, could also be used with other nectarine cultivars and/or different stone fruits.

## Figures and Tables

**Table 1:** Dunnett’s test controlling false discovery rate at 0.01 level: volatile compounds detected in nectarines cv Big Bang at 0 days (fresh) and during storage (3, 7 and 10 days) at two temperatures (4 and 8 °C) and their identification codes.

VOCs	Code	FRESH	4 °C			8 °C		
			3 d	7 d	10 d	3 d	7 d	10 d
Ethyl acetate	E1	nd	nd (ns)	nd (ns)	3.1 (s)	nd (ns)	9.9 (s)	132.6 (s)
Ethyl propanoate	E2	nd	nd (ns)	nd (ns)	nd (ns)	nd (ns)	nd (ns)	1.7 (s)
Propyl acetate	E3	nd	nd (ns)	nd (ns)	nd (ns)	nd (ns)	nd (ns)	1.3 (s)
2-Methylpropyl acetate	E4	nd	nd (ns)	nd (ns)	nd (ns)	nd (ns)	nd (ns)	6.6 (s)
Ethyl butyrate	E5	nd	nd (ns)	nd (ns)	nd (ns)	nd (ns)	0.2 (s)	0.7 (s)
Methyl caproate	E6	nd	0.8 (s)	0.6 (s)	nd (ns)	0.8 (s)	0.8 (s)	nd (ns)
Hexyl acetate	E7	64.3	18.5 (s)	176.7 (s)	6.6 (s)	10.6 (s)	6 (s)	2.7 (s)
3-Hexen-1-ol acetate	E8	124.2	45.8 (s)	37.8 (s)	27.4 (s)	20.1 (s)	12.3 (s)	12.5 (s)
2-Hexen-1-ol acetate	E9	66.8	28.1 (s)	173.1 (s)	30.5 (s)	24.5 (s)	21.5 (s)	29.6 (s)
Ethyl octanoate	E10	3.9	nd (s)	nd (s)	1.9 (s)	nd (s)	2.6 (s)	4.4 (ns)
*cis*-3-Hexenyl isobutyrate	E11	1.1	nd (s)	nd (s)	nd (s)	nd (s)	nd (s)	nd (s)
2-Hexenyl butanoate	E12	2.4	0.5 (s)	1.6 (s)	nd (s)	1.2 (s)	1.0 (s)	nd (s)
Hexyl caproate	E13	1.7	nd (s)	nd (s)	nd (s)	nd (s)	nd (s)	nd (s)
*cis*-3-Hexenyl hexanoate	E14	0.7	nd (s)	nd (s)	nd (s)	nd (s)	nd (s)	nd (s)
*cis*-2-Hexenyl hexanoate	E15	1.5	nd (s)	nd (s)	nd (s)	nd (s)	nd (s)	nd (s)
3-Pentanone	K1	7.7	2.7 (s)	2.5 (s)	4.4 (s)	3.1 (s)	2.4 (s)	5.6 (s)
1-Penten-3-one	K2	1.0	1.6 (ns)	1.1 (ns)	1.7 (s)	2.1 (s)	0.9 (ns)	2.5 (s)
3-Octanone	K3	5.3	6.6 (s)	6.6 (s)	6.5 (s)	6.6 (s)	7.4 (s)	8.0 (s)
1-Octen-3-one	K4	nd	0.8 (s)	nd (ns)	nd (ns)	0.8 (s)	nd (ns)	nd (ns)
Hexanal	Ald1	75.8	46.1 (s)	29.5 (s)	61.4 (s)	63.1 (s)	26.3 (s)	44.3 (s)
3-Hexenal	Ald2	3.0	3.6 (ns)	2.5 (ns)	4.7 (s)	3.5 (ns)	3 (ns)	6.1 (s)
Heptanal	Ald3	nd	nd (ns)	nd (ns)	1.1 (s)	nd (ns)	nd (ns)	1.4 (s)
2-Hexenal	Ald4	156.7	325.3 (s)	235.1 (s)	352.1 (s)	459.1 (s)	259.3 (s)	626.2 (s)
Octanal	Ald5	3.4	1.4 (s)	nd (s)	1.7 (s)	nd (s)	nd (s)	nd (s)
*trans*-2-Heptenal	Ald6	4.5	2.4 (s)	nd (s)	nd (s)	2.5 (s)	nd (s)	3.1 (s)
2-Octenal	Ald7	nd	1.4 (s)	nd (ns)	nd (ns)	1.7 (s)	nd (ns)	nd (ns)
Decanal	Ald8	nd	nd (ns)	nd (ns)	0.8 (s)	nd (ns)	nd (ns)	1.6 (s)
Benzaldehyde	Ald9	nd	0.6 (s)	1.4 (s)	1.2 (s)	1.5 (s)	0.7 (s)	3.4 (s)
1-Penten-3-ol	Al1	16.8	5.7 (s)	5.8 (s)	9.2 (s)	5.4 (s)	5.3 (s)	9.0 (s)
*cis*-2-Penten-1-ol	Al2	nd	nd (ns)	3.9 (s)	4.8 (s)	4.1 (s)	2.6 (s)	4.4 (s)
1-Hexanol	Al3	353.0	110 (s)	155 (s)	57.4 (s)	128.1 (s)	41.5 (s)	71.6 (s)
*cis*3-Hexenol	Al4	11.4	3.6 (s)	5.9 (s)	4.9 (s)	4.4 (s)	2.6 (s)	6.4 (s)
*trans*-3-Hexenol	Al5	153.3	50.7 (s)	14.2 (s)	24.4 (s)	32.5 (s)	13.5 (s)	34.1 (s)
2-Hexen-1-ol	Al6	335.7	257.8 (s)	230.7 (s)	207.6 (s)	251.4 (s)	122.4 (s)	299.5 (s)
1-Octen-3-ol	Al7	1.7	1.1 (s)	0.9 (s)	0.9 (s)	1.0 (s)	0.6 (s)	14.6 (s)
2-Ethylhexanol	Al8	nd	nd (ns)	1.2 (s)	1.2 (s)	nd (ns)	nd (ns)	1.7 (s)
1-Octanol	Al9	1.1	0.4 (s)	0.3 (s)	0.5 (s)	0.1 (s)	0.3 (s)	nd (s)
2-Furanmethanol	Al10	nd	nd (ns)	nd (ns)	nd (ns)	0.1 (s)	nd (ns)	nd (ns)
Limonene	T1	1.8	nd (s)	nd (s)	nd (s)	nd (s)	nd (s)	nd (s)
Linalool	T2	1.9	0.5 (s)	nd (s)	nd (s)	0.3 (s)	nd (s)	nd (s)
*α*-Farnesene	T3	1.5	0.3 (s)	0.3 (s)	nd (s)	0.3 (s)	0.2 (s)	nd (s)
Pentanoic acid	Ac1	nd	0.7 (ns)	0.7 (ns)	0.5 (ns)	0.6 (ns)	0.9 (ns)	2.5 (ns)
Hexanoic acid	Ac2	nd	nd (ns)	nd (ns)	nd (ns)	0.2 (s)	nd (ns)	nd (ns)
2-Ethylfuran	O1	nd	0.2 (s)	nd (ns)	0.4 (s)	0.1 (s)	nd (ns)	nd (ns)
Heptadecane	O2	nd	nd (ns)	0.1 (ns)	0.2 (s)	0.2 (s)	0.2 (s)	nd (ns)
*γ-*Caprolactone	O3	nd	0.8 (s)	0.9 (s)	1.7 (s)	0.8 (s)	1.1 (s)	4.4 (s)

Mean values of three samples; nd means not detected, s means significant and ns means not significant.

**Table 2:** Dunnett’s test controlling false discovery rate at level 0.05: phenolic compounds detected in nectarines cv “Big Bang” at 0 days (fresh) and during storage (3, 7 and 10 days) at two temperatures (4 and 8 °C) and their identification codes.

Phenolic Compounds	Code	FRESH	4 °C			8 °C		
			3 d	7 d	10 d	3 d	7 d	10 d
Neochlorogenic acid	P1	12.7	10.6 (ns)	33.7 (s)	13.1 (ns)	23.5 (s)	17.9 (s)	20.8 (s)
Cryptochlorogenic acid	P2	8.8	9.3 (ns)	7.9 (ns)	10.4 (ns)	5.0 (s)	10.9 (s)	7.8 (ns)
Chlorogenic acid	P3	33.3	37.5 (s)	49.9 (s)	24.4 (s)	41.2 (s)	26.3 (s)	35.5 (ns)
Cyanidin-3-O-glucoside	P4	45.6	22.8 (s)	41.3 (s)	22.1 (s)	38.2 (s)	17.2 (s)	21.8 (s)
Rutin	P5	25.6	31.9 (s)	22.2 (s)	30.9 (s)	16.8 (s)	26.8 (ns)	19.3 (s)

Mean values of three samples; s means significant and ns means not significant.

## Data Availability

The original contributions presented in the study are included in the article/Supplementary Materials, further inquiries can be directed to the corresponding author.

## Supplementary Materials

The following supporting information can be downloaded at: https://www.mdpi.com/article/10.3390/foods13071047/s1, Table S1: Volatile organic compounds (VOCs) detected in nectarine cv Big Bang and their identification codes.
