# Supplementary material for: Volatile Metabolites to Assess the Onset of Chilling Injury in Fresh-Cut Nectarines
Source: Foods. 2024 Mar 29;13(7):1047. doi: 10.3390/foods13071047 (PMC11011915; doi:10.3390/foods13071047)
Supplement: Supplementary file 1 [file foods-13-01047-s001.zip › foods-2938917-supplementary.pdf]

Volatile Metabolites to Assess the Onset of Chilling Injury in Fresh-Cut Nectarines

Table S1. - Volatile organic compounds (VOCs) detected in nectarine cv Big Bang and their identification codes.

Metabolite	Code	[a]Ri$_t$/RI$_{sp}$	[b]ID	Metabolite	Code	[a]Ri$_t$/RI$_{sp}$	[b]ID
**Esters**				**Aldehydes**			
Ethyl acetate	E1	935/921	RI/MS/S	Hexanal	Ald1	1076/1075	RI/MS/S
Ethyl propionate	E2	973/971	RI/MS/S	3-Hexenal	Ald2	1137/1134	RI/MS/S
Propyl acetate	E3	984/982	RI/MS/S	Heptanal	Ald3	1191/1190	RI/MS/S
2-Methylpropyl acetate	E4	1013/1012	RI/MS/S	2-Hexenal	Ald4	1227/1224	RI/MS/S
Ethyl butyrate	E5	1033/1032	RI/MS/S	Octanal	Ald5	1292/1291	RI/MS/S
Methyl hexanoate	E6	1191/1190	RI/MS/S	*trans*-2-Heptenal	Ald6	1329/1326	RI/MS/S
Hexyl acetate	E7	1274/1275	RI/MS/S	2-Octenal	Ald7	1430/1430	RI/MS/S
trans-3-Hexenyl acetate	E8	1316/1316	RI/MS/S	Decanal	Ald8	1505/1505	RI/MS/S
cis-2-Hexenyl acetate	E9	1333/1331	RI/MS/S	Benzaldehyde	Ald9	1529/1529	RI/MS/S
Ethyl octanoate	E10	1430/1430	RI/MS/S	**Ketones**			
cis-3-Hexenyl isobutyrate	E11	1456/1482	RI/MS/S	3-Pentanone	K1	988/986	RI/MS/S
2-Hexenyl butyrate	E12	1471/1466	RI/MS/S	1-Penten-3-one	K2	1024/1024	RI/MS/S
Hexyl octanoate	E13	1609/1608	RI/MS/S	3-Octanone	K3	1258/1258	RI/MS/S
cis-3-Hexenyl hexanoate	E14	1656/1654	RI/MS/S	1-Octen-3-one	K4	1305/1305	RI/MS/S
cis-2-Hexenyl hexanoate	E15	1671/1653	RI/MS	**Terpenes**			
**Alcohols**				dl-Limonene	T1	1192/1190	RI/MS/S
1-Penten-3-ol	Al1	1171/1170	RI/MS/S	Linalool	T2	1548/1548	RI/MS/S
cis-2-Penten-1-ol	Al2	1324/1322	RI/MS	α-Farnesene	T3	1749/1749	RI/MS
1-Hexanol	Al3	1355/1354	RI/MS/S	**Acids**			
cis-3-Hexen-1-ol	Al4	1363/1363	RI/MS/S	Pentanoic acid	Ac1	1670/1686	RI/MS/S
trans-3-Hexen-1-ol	Al5	1382/1381	RI/MS/S	Hexanoic acid	Ac2	1847/1847	RI/MS/S
trans-2-Hexen-1-ol	Al6	1403/1403	RI/MS/S	**Others**			
1-Octen-3-ol	Al7	1449/1449	RI/MS/S	2-Ethylfuran	O1	970/965	RI/MS/S
2-Ethyl-1-hexanol	Al8	1492/1491	RI/MS/S	Heptadecane	O2	1701/1700	RI/MS/S
1-Octanol	Al9	1560/1560	RI/MS/S	γ-Caprolactone	O3	1704/1703	RI/MS/S
2-Furanmethanol	Al10	1663/1663	RI/MS/S				

[a]RI$_t$: Relative retention indices on polar column reported in literature by www.pherobase.com; www. flavornet.org; www.ChemSpider.com; webbook.nist.gov; RI$_{sp}$: Relative retention indices calculated against n-alkanes (C_8-C_{40}) on HP-Innowax column; [b]Identification method as indicated by the following: RI: Kovats retention index on a on HP-Innowax column; MS: NIST and Wiley libraries spectra; S: co-injection with authentic standard compounds on the HP-Innowax column
